# Optimization of Enzymatic Extraction and Vacuum‐Assisted Evaporation Parameters for Banana Syrup Production

**DOI:** 10.1155/ijfo/8891536

**Published:** 2025-10-08

**Authors:** Narathip Sujinda, Natthiya Chaichana, Thanapon Saengsuwan, Jaturapatr Varith

**Affiliations:** ^1^ Faculty of Agriculture at Kamphaeng Saen, Kasetsart University, Kamphaeng Saen Campus, Nakhon Pathom, Thailand, ku.ac.th; ^2^ Science Program, Faculty of Education, Chiang Rai Rajabhat University, Chiang Rai, Thailand; ^3^ Energy Engineering and Electric Technology Program, Faculty of Industrial Technology, Chiang Rai Rajabhat University, Chiang Rai, Thailand; ^4^ Food Engineering Program, Faculty of Engineering and Agro-Industry, Maejo University, Chiang Mai, Thailand, mju.ac.th

**Keywords:** banana syrup, cellulase, enzymatic extraction, pectinase, vacuum evaporation

## Abstract

Bananas are a widely cultivated fruit with significant nutritional and economic value; however, postharvest losses remain a major concern. This study is aimed at developing an optimized process for enzymatic extraction to produce banana syrup. This requires an assessment of the influence exerted by different concentrations of pectinase and cellulase, as well as the effects of incubation time and temperature. To optimize the conditions, a Box–Behnken design (BBD) was used, while juice yield and soluble solids recovery were modeled using response surface methodology (RSM). The results indicate that a combination of 0.39% pectinase, 0.46% cellulase, 49.8°C incubation temperature, and 129.6 min of incubation time maximized juice yield (71.4%) and soluble solids recovery (22.3%). The quality of syrup was maintained through the use of vacuum evaporation at 40°C and 100 kPa, ensuring the preservation of viscosity and clarity while minimizing browning. The results imply that both enzymatic extraction and vacuum evaporation offer potential for application within the food sector as a means to enhance production, cut wastage, and promote greater efficiency.

## 1. Introduction

Bananas are a very important agricultural product that is exported worldwide due to their nutritional value, flavor, and range of applications in different food products [1]. With an estimated global production exceeding 150 million tons, bananas serve as a key agricultural commodity, especially within the economies of tropical or subtropical countries [2]. However, challenges facing the industry include overproduction, physical damage to the product in transit, and a lack of adequate infrastructure for processing. Innovative approaches are therefore necessary in order to address the losses incurred after harvesting and to add value to surplus banana crops.

One way to add value is to produce banana syrup, which may find varied applications within the food sector due to its natural sweetness, attractive taste, odor, and texture and advantages for enhanced human health [3]. The difficulty, however, lies in extracting the banana syrup since the banana matrix has a complex structure comprising pectin, cellulose, and various other polysaccharides. Pectin and starch have the effect of increasing the viscosity and turbidity of banana juice, so in order to clarify the extracted product, enzymatic treatment is required. These polysaccharides can be minimized through the use of pectinase [4], leading to enhanced clarity of the juice, thus easing the ongoing production process. Furthermore, the various polysaccharides can also obstruct the release of soluble solids, making it necessary to develop an extraction approach capable of improving the quality and quantity of the syrup yield. Earlier research has revealed improvements in the efficiency of juice extraction through enzymatic treatment involving cellulase and pectinase, since these agents can break down the walls of plant cells, thus releasing intracellular components. Singh et al. [5] demonstrated that pectinase‐assisted enzymatic extraction improved juice recovery and reduced viscosity in sapodilla (*Manilkara achras* L.) juice, further highlighting the potential of enzymatic hydrolysis in fruit processing. In addition, it was observed by Laskar et al. [6] that the yield for banana juice can be enhanced by citric acid, hot water, and enzymatic hydrolysis, leading to a higher quality product. This demonstrates once again the benefits of employing a multistage process for extraction.

While extensive research has focused on banana juice extraction [7], the production of banana syrup requires additional processing steps to achieve the desired concentration and stability. Thermal processing methods used for syrup concentration must balance efficiency with quality preservation to prevent undesirable alterations which affect the flavor, the color, or the nutritional value of the product [8]. Vacuum evaporation, on the other hand, has been shown to effectively concentrate fruit‐based products while minimizing heat‐induced deterioration, preserving color and maintaining nutritional integrity [1, 9, 10]. Compared to conventional methods, vacuum evaporation does not require high temperatures, thus avoiding the problem of bioactive compounds undergoing degradation as a consequence of the heat. In turn, this allows the original flavor to be maintained. Research into pomegranate juice concentration found that rotary vacuum evaporation minimized color changes and retained sensory quality better than atmospheric heating [10]. According to Bozkir and Baysal [9], in another study involving apple juice, phenolic compounds and color parameters were found to be well preserved when a vacuum microwave evaporation technique was employed. Earlier findings are augmented by the current work, which makes use of enzymatic extraction in combination with vacuum evaporation in order to optimize the production conditions to efficiently and sustainably obtain high quality banana syrup. Although many studies have focused upon enzymatic extraction in the context of producing fruit juice [4, 5], the literature rarely mentions this approach in conjunction with banana syrup. The optimization of enzymatic hydrolysis alongside vacuum evaporation might therefore serve to improve the efficiency of extraction as well as the stability of the resulting product. To ensure optimal enzyme‐assisted extraction of banana juice, response surface methodology (RSM) is a valuable tool for identifying the most effective combination of enzymatic and thermal parameters. RSM enables efficient modeling of factor interactions, such as enzyme concentrations, temperature, and incubation time, while minimizing the number of experimental trials. This approach has been effectively applied in optimizing enzymatic extraction processes to improve juice yield and clarity [11, 12] In this study, RSM was applied specifically to optimize the enzymatic extraction step, which plays a crucial role in improving both the quality and efficiency of banana syrup production.

This study has the principal goal of optimizing the parameters which are set to perform an enzymatic extraction procedure that will maximize both the juice yield and recovery of soluble solids. This necessitates experimentation to determine the ideal conditions for incubation time and temperature, and the concentrations of the enzymes employed. Furthermore, the influence upon the quality of the syrup when using vacuum evaporation is also investigated, with comparisons drawn to traditional techniques in order to establish which approach might offer superior outcomes. Given the increasing focus on sustainable food processing and circular economy principles, enzymatic extraction offers a greener alternative, reducing energy consumption and minimizing by‐product waste [13]. This study seeks a means of adding significant value to the banana, since the syrup is a high‐value product offering economic and environmental sustainability if these techniques can be applied on an industrial scale in the processing of bananas. However, there remains a research gap in the integrated application of enzyme‐assisted extraction and vacuum‐assisted evaporation specifically for banana syrup production. Most previous studies have addressed extraction and concentration separately without optimizing their combination. It is therefore proposed that optimizing enzyme‐assisted extraction using RSM, followed by evaluation of different vacuum evaporation conditions, can enhance juice yield, soluble solids recovery (SSR), and overall syrup quality in a more energy‐efficient and sustainable manner.

## 2. Material and Methods

### 2.1. Raw Material Preparation

The bananas (*Musa sapientum L.*) used for syrup production were selected at a ripeness stage of 7–8 based on the peel color index [14], where the peel exhibited a yellow hue with visible brown speckles or large brown patches. Whole bananas (with peel) were steamed at 90°C for 14 min to inactivate browning enzymes and preserve pulp structure, as recommended by Wohlt et al. [15]. After cooling, the peel was removed, and only the pulp was finely processed and stored at −20°C until further processing.

### 2.2. Experimental Design and Optimization

The optimization of banana juice extraction was conducted using the Box–Behnken design (BBD) [6, 16] to evaluate the effects of four key factors: pectinase concentration, cellulase concentration, incubation temperature, and incubation time. Each factor was studied at three levels: pectinase concentration (0.1%, 0.3%, and 0.5%), cellulase concentration (0.1%, 0.3%, and 0.5%), incubation temperature (40°C, 50°C, and 60°C), and incubation time (60, 120, and 180 min). The BBD design included 27 experimental runs, incorporating replicates at the center points to enhance statistical accuracy and reliability. Arranging the experiment in this manner permitted the evaluation of quadratic effects and interactions among the various factors, thus enabling the establishment of optimized parameters to maximize banana juice yield, SSR, and clarity. RSM was employed to examine the links between the independent variables and response values for the experimental data. Variables deemed to be significant (*p* < 0.05) could then be selected for the second‐order polynomial model shown below as:

(1) Y=b0+∑bixi+∑biixi2+∑bijxixj,

in which the response value is indicated by *Y*, the intercept is *b*_0_, the linear effects coefficients are represented by *b*_*i*_, the squared effects coefficients are shown as *b*_*i* *i*_, and *b*_*i* *j*_ indicates the coefficients associated with the effects of interaction between independent variables *x*_*i*_ and *x*_*j*_.

Factor interactions could be visualized following the generation of contour and 3D surface plots, enabling the optimal processing parameters to be established. This approach provided a systematic and robust framework for optimizing the banana juice extraction process, ensuring enhanced process efficiency and juice quality.

### 2.3. Validation of Optimized Process Parameters

Having obtained a prediction of the optimal extraction process conditions using RSM models, the procedure was performed in vivo to assess the reliability of the model. The incubation time and temperature are clearly defined in the optimal conditions, along with the precise concentrations required for cellulase and pectinase. Under such conditions, the juice yield and recovery of soluble solids should be maximized. Under experimental conditions, the juice yield and recovery of soluble solids were measured and subsequently compared to the values predicted by the RSM model. Any differences were then calculated via the formula given below:

(2) Percentage Difference=Experimental Value−Predicted ValuePredicted Value×100.

### 2.4. Experimental Procedure

#### 2.4.1. Banana Juice Extraction

Frozen banana pulp was thawed and treated with specific concentrations of pectinase (Pectinex Ultra SP‐L, 3300 PGNU/g; Novozymes A/S, Denmark) and cellulase (Celluclast 1.5 L, ≥ 70 FPU/mL; Novozymes A/S, Denmark) according to the BBD matrix. The enzymatic treatment was conducted in a controlled water bath at designated temperatures (40°C, 50°C, and 60°C) and incubation times (60, 120, and 180 min). Following the incubation period, the samples were immersed in boiling water (~100°C) for a period of 10 min in order to halt the enzyme activity, followed by allowing the samples to cool down quickly via immersion in an ice bath (0°C–4°C) [17]. The juice was then separated using a refrigerated centrifuge (VARISPIN 15R) at 8000 rpm and 4°C for 25 min. The extracted juice was analyzed for key quality parameters, including juice yield and SSR, as described in Equations (3) and (4), respectively.

(3) Juice yeild %=Weight of extracted juice Weight of banana pulp×100.

(4) SSR %=Weight of juice yield×TSS of juiceWeight of banana pulp×100.

#### 2.4.2. Banana Syrup Production

After juice extraction under optimal conditions, the banana syrup was concentrated using vacuum evaporation at specific predetermined temperatures (40°C, 50°C, and 60°C) and vacuum levels (0, 60, 80, and 100 kPa). The evaporation process was continued until 60% of the initial water content was removed, ensuring a controlled concentration process. The final syrup was evaluated for its physicochemical properties, including total soluble solids (TSS) (°Brix), color (*L* ^∗^, *a* ^∗^, and *b* ^∗^), clarity, and viscosity.

### 2.5. Quality Assessment

In assessing the quality of the banana juice, the key criteria were the yield, recovery of soluble solids, and clarity. In the case of banana syrup quality, the criteria comprised color, yield, clarity, viscosity, and TSS (°Brix). The use of these criteria supported the investigation of the processing parameters necessary in order to optimize the product quality.

#### 2.5.1. Color

A colorimeter (HunterLab MiniScan XE Plus) was used to measure the color (CIE *L* ^∗^, *a* ^∗^, and *b* ^∗^) of the banana syrup. As demonstrated by Julai et al. [18] in date syrup and by Handique et al. [7] in banana juice, accurate color measurement is essential for evaluating enzymatic browning and thermal degradation during processing.

#### 2.5.2. Clarity

Clarity (% transmittance at 660 nm) was assessed using a spectrophotometer (Shimadzu UV‐1800). This parameter was particularly relevant for banana syrup; clarity was analyzed to assess the degree of suspended solids removal and thermal stability [11].

#### 2.5.3. Viscosity

The viscosity (mPa·s) of banana syrup was measured using a rotational viscometer (Brookfield DV2T) equipped with an RV‐3 spindle, operated at 50 rpm. A comparable method was employed by Handique et al. [7] to assess the flow behavior of enzyme‐treated banana juice.

#### 2.5.4. SSR

SSR (percent) was determined using a Hand Refractometer (Hanna Instruments HI96801) and was particularly relevant to banana juice to assess the efficiency of enzymatic extraction [11, 18].

#### 2.5.5. TSS

TSS (degree Brix) in both banana juice and syrup was measured using the same refractometer method. This parameter was essential for ensuring proper concentration levels in syrup production, as also emphasized by Rosemary et al. [19], Julai et al. [18], and Dowerah et al. [11] in their studies on fruit‐based syrups and juices.

By designing this study to focus on certain key parameters, it was possible to draw comparisons among various combinations of processing conditions in order to determine how the quality of the banana juice and syrup would be affected. With these data, it would then be feasible to optimize the production conditions to deliver a superior product with ideal characteristics.

### 2.6. Statistical Analysis

A RSM model was created to make predictions based upon the available data in order to identify the optimal processing conditions. To explain the relationships linking independent variables and response values, a second‐order polynomial equation was produced. Those models capable of delivering a level of confidence exceeding 95% (*p* ≤ 0.05) and an *R* ^2^ value exceeding 0.75 were selected for further analysis. To visualize the effects of interactions among different factors, contour plots were produced. This allowed a deeper understanding of the exact nature of how incubation time and temperature, along with the concentration of the enzymes, could affect the quality and efficiency of juice extraction. For analysis of significant differences in the evaporation conditions when producing banana syrup, Duncan′s multiple range test (*p* < 0.05) was utilized. This statistical approach ensured that the optimal processing conditions were validated, enhancing the quality, efficiency, and consistency of banana syrup production.

## 3. Results and Discussion

### 3.1. Optimal Conditions for Banana Juice Extraction

Through the process of optimizing the extraction of banana juice, it could be determined that the concentration of pectinase (*x*_1_), concentration of cellulase (*x*_2_), temperature (*x*_3_), and incubation time (*x*_4_) significantly influenced key responses, particularly for banana juice yield and SSR (Table 1). Table 2 shows the results for the ANOVA, which indicate the significance of the effects of these variables upon the efficiency of extraction. In particular, the concentrations of pectinase and cellulase had the greatest influence upon juice yields and the recovery of soluble solids (*p* ≤ 0.05). Additionally, the interaction effects among these factors played a crucial role in optimizing enzymatic hydrolysis efficiency.

**Table 1:** Box–Behnken design: Independent and dependent variables.

**Exp. no.**	**Pectinase (%)**	**Cellulase (%)**	**Temperature (°C)**	**Incubation time (min)**	**Juice yield (%)**	**SSR (%)**
1	0.3	0.5	60	120	65.0	20.9
2	0.1	0.3	50	60	54.4	17.3
3	0.3	0.1	50	60	57.0	17.3
4	0.1	0.3	60	120	59.9	18.7
5	0.5	0.5	50	120	70.4	23.4
6	0.5	0.1	50	120	55.1	17.9
7	0.3	0.3	40	60	54.6	16.1
8	0.5	0.3	60	120	55.0	17.7
9	0.3	0.5	50	60	60.8	18.9
10	0.3	0.3	40	180	52.1	17.3
11	0.3	0.1	50	180	56.0	17.9
12	0.3	0.5	50	180	64.9	20.6
13	0.1	0.1	50	120	58.3	19.1
14	0.3	0.3	60	180	57.6	18.0
15	0.1	0.5	50	120	63.1	19.7
16	0.3	0.3	50	120	71.2	22.3
17	0.1	0.3	40	120	51.8	16.1
18	0.1	0.3	50	180	55.0	17.1
19	0.3	0.1	40	120	51.8	16.5
20	0.3	0.3	50	120	71.0	22.7
21	0.3	0.5	40	120	63.4	19.1
22	0.3	0.3	60	60	54.2	17.0
23	0.3	0.1	60	120	55.1	17.1
24	0.3	0.3	50	120	72.0	22.6
25	0.5	0.3	50	60	54.4	16.8
26	0.5	0.3	40	120	59.1	19.1
27	0.5	0.3	50	180	60.6	18.9

**Table 2:** Analysis of variance for response variables.

**Source**	**Juice yield**	**SSR**
Model	994.97 ^∗^	112.28 ^∗^
Pectinase (*x*_1_)	12.2 ^∗^	2.80 ^∗^
Cellulase (*x*_2_)	245.71 ^∗^	23.52 ^∗^
Temperature (*x*_3_)	16.33 ^∗^	2.25 ^∗^
Incubation time (*x*_4_)	9.72 ^∗^	3.41 ^∗^
${x_{1}}^{2}$	222.74 ^∗^	17.52 ^∗^
${x_{2}}^{2}$	64.87 ^∗^	5.20 ^∗^
${x_{3}}^{2}$	399.05 ^∗^	42.94 ^∗^
${x_{4}}^{2}$	376.32 ^∗^	44.47 ^∗^
*x*_1_ *x*_2_	27.56 ^∗^	6.00 ^∗^
*x*_1_ *x*_3_	37.21 ^∗^	4.00 ^∗^
*x*_1_ *x*_4_	7.84 ns	1.32 ^∗^
*x*_2_ *x*_3_	0.72 ns	0.36 ns
*x*_2_ *x*_4_	6.5 ns	0.30 ns
*x*_3_ *x*_4_	8.7 ^∗^	0.01 ns
Lack of fit	19.43 ns	2.48 ns
*R* ^2^	0.980	0.978
Adj. *R* ^2^	0.957	0.952

*Note:* Significance levels:  ^∗^ = *p* ≤ 0.05, ns = not significant.

The interaction between pectinase and cellulase concentrations (*x*_1_ *x*_2_) significantly influenced both juice yield and SSR, as depicted in Figures 1a and 2a. According to this interaction, cellulase further breaks down cellulose and hemicellulose to improve juice yield and the extraction of soluble solids, whereas pectinase mainly breaks down pectin‐rich structures to facilitate the release of intracellular components. Higher extraction efficiency was promoted by improved hydrolysis and decreased juice viscosity brought about by increasing the amounts of both enzymes. On the other hand, high enzyme concentrations may cause overhydrolysis, which would make the juice more viscous and decrease filtration effectiveness. This conclusion is consistent with earlier research on enzymatic processing of fruit juice, indicating that combinations of pectinase and cellulase markedly enhanced yield and decreased processing duration [3, 5].

Figure 1Effects of enzyme concentrations, time, and temperature on banana juice yield. (a) pectinase and cellulase, (b) pectinase and temperature, (c) cellulase and temperature, (d) cellulase and time, (e) pectinase and time, and (f) temperature and time.

**Figure:**
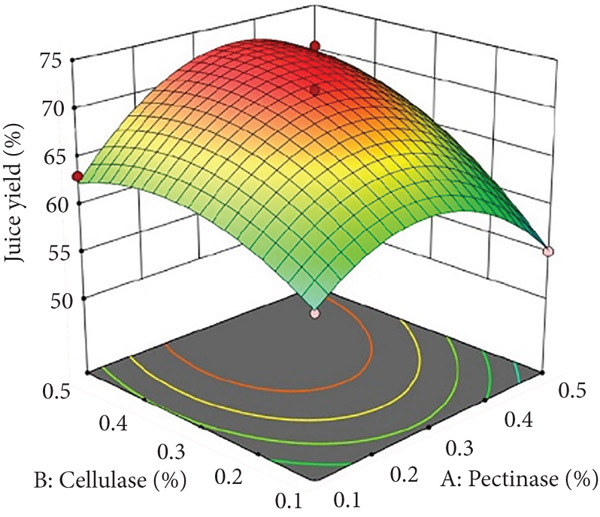
(a)

**Figure:**
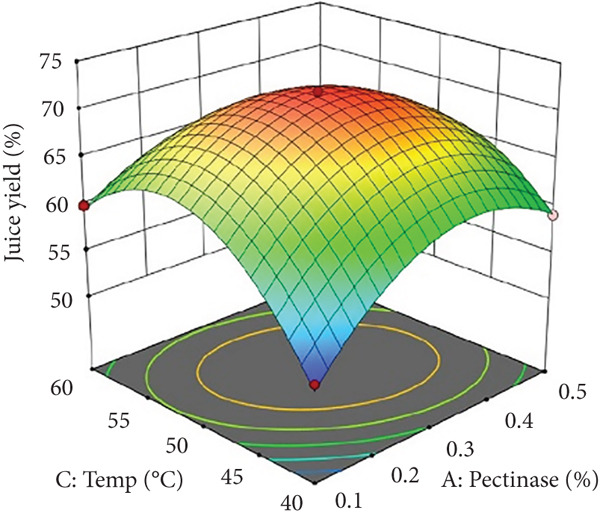
(b)

**Figure:**
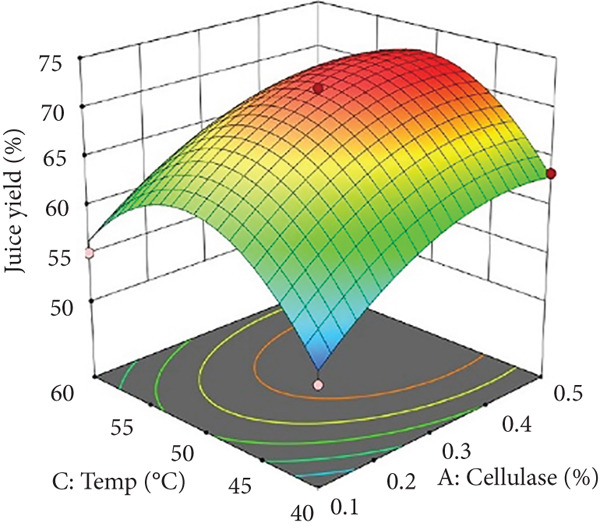
(c)

**Figure:**
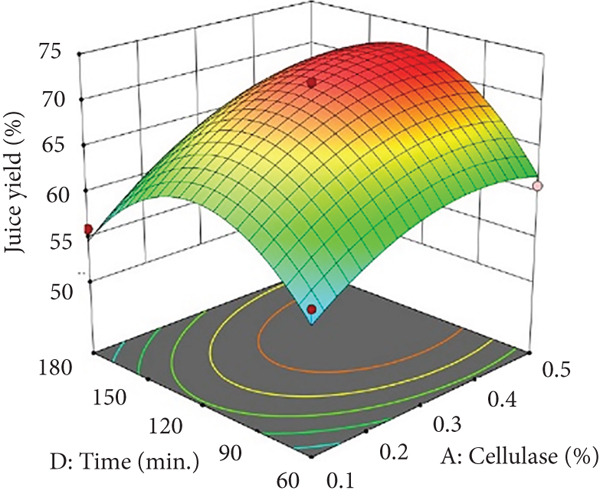
(d)

**Figure:**
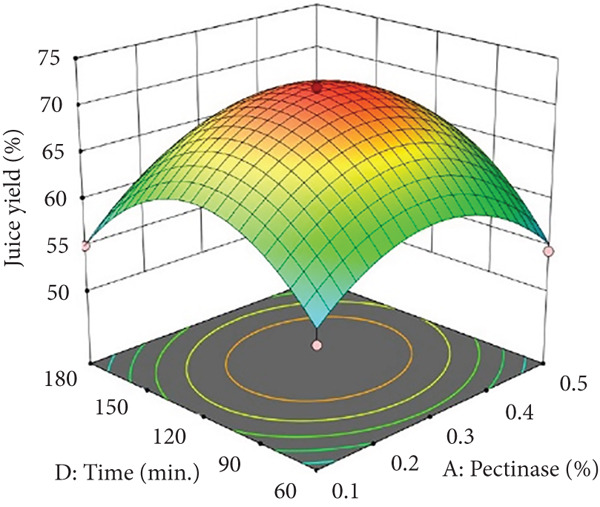
(e)

**Figure:**
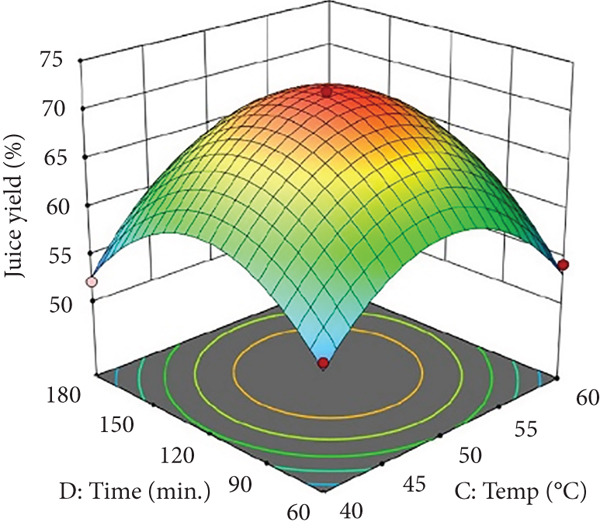
(f)

Figure 2Effects of enzyme concentrations, time, and temperature on soluble solids recovery (SSR). (a) pectinase and cellulase, (b) pectinase and temperature, (c) cellulase and temperature, (d) cellulase and time, (e) pectinase and time, and (f) temperature and time.

**Figure:**
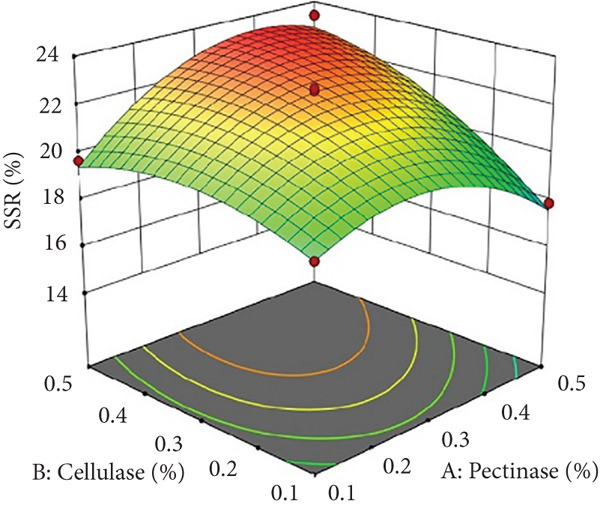
(a)

**Figure:**
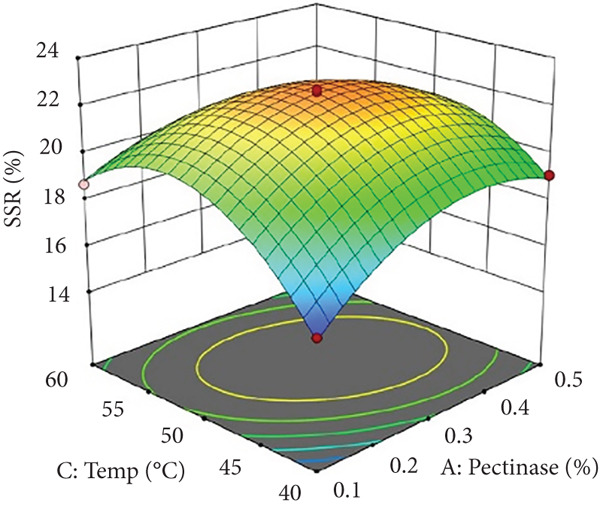
(b)

**Figure:**
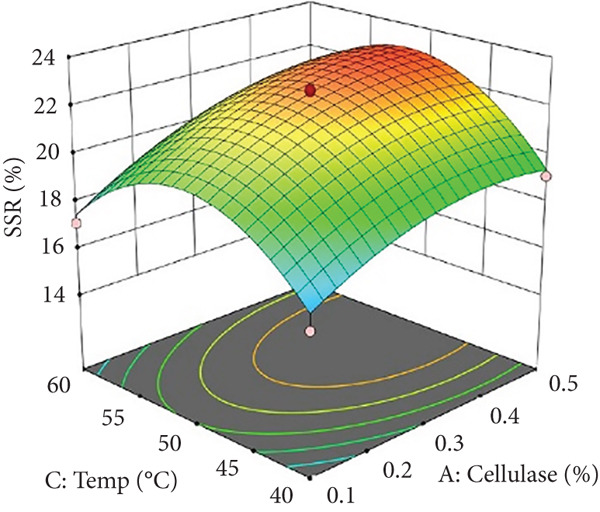
(c)

**Figure:**
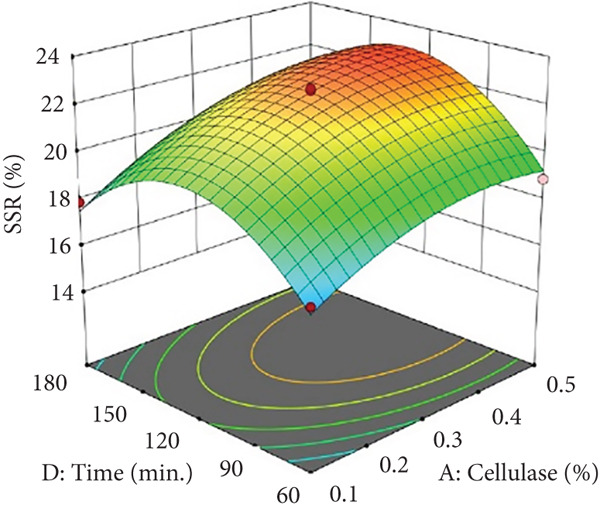
(d)

**Figure:**
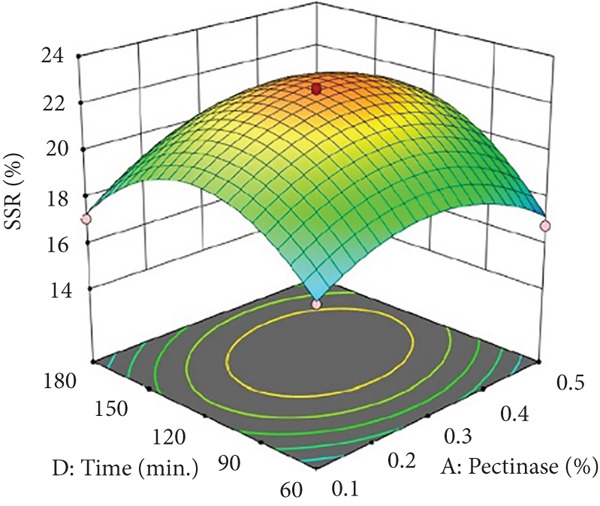
(e)

**Figure:**
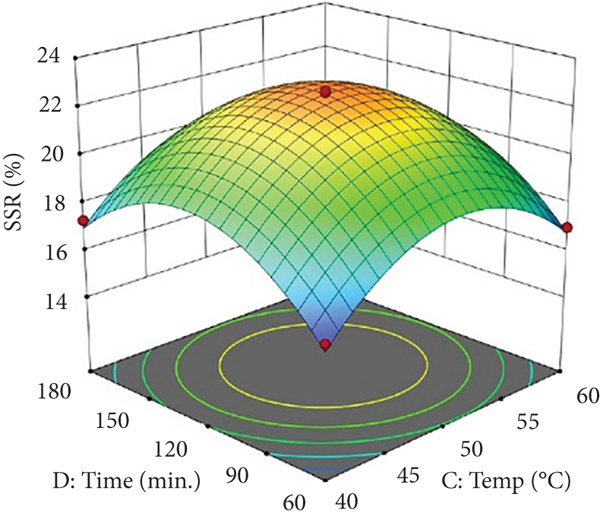
(f)

Figures 1b and 2b show that the relationship between the amount of pectinase and the temperature (*x*_1_ *x*_3_) had a big effect on both the juice yield and the SSR. At temperatures up to 50°C, pectinase activity was enhanced, facilitating efficient breakdown of pectin and reducing juice viscosity, thereby increasing juice yield and soluble solids extraction. However, beyond 50°C, enzyme denaturation occurred, leading to a decline in juice yield and SSR due to loss of enzymatic activity. These results align with prior research demonstrating that pectinase operates effectively at temperatures between 45°C and 50°C, although it exhibits instability at elevated temperatures [4]. Maintaining an appropriate temperature range is crucial for preserving enzyme activity and optimizing extraction efficiency. Moreover, although the interactions between cellulase concentration and temperature (*x*_2_ *x*_3_), and incubation time (*x*_2_ *x*_4_), were not statistically significant (*p* > 0.05; Table 2), their trends remain noteworthy based on the response surface plots. As illustrated in Figures 1c,d and 2c,d, cellulase contributed to the breakdown of cellulose and hemicellulose, particularly at moderate temperatures (~50°C) and extended incubation durations (120–180 min), resulting in improved juice yield and SSR. However, under prolonged incubation or excessive thermal conditions, enzyme degradation may occur. Although the viscosity of banana juice was not measured in this study, previous research has indicated that elevated viscosity can hinder filtration efficiency under such conditions [3–5]. The correlation between incubation temperature and duration (*x*_3_ *x*_4_) significantly influenced juice output (Figures 1f and 2f). Prolonged incubation at moderate temperatures (50°C) provided adequate time for enzymatic hydrolysis, enhancing juice extraction efficiency. At increased temperatures (surpassing 50°C) and prolonged incubation periods, enzyme degradation transpired, leading to a reduction in yield. This conclusion is supported by previous studies indicating that enzymatic activity declines due to thermal instability caused by prolonged heat exposure [7]. These findings highlight the importance of adjusting incubation time and temperature to avert enzyme deactivation while ensuring complete hydrolysis of banana cell wall components. The interaction between pectinase concentration and incubation time (*x*_1_ *x*_4_) significantly influenced SSR (Figures 1e and 2e). Pectinase, known for its role in breaking down pectin‐rich components, showed improved activity with extended incubation times, as prolonged exposure allowed the enzyme to fully hydrolyze pectin structures, leading to enhanced release of soluble solids. Short incubation periods (≤ 60 min) caused partial hydrolysis, whereas extremely extended incubation times (≥ 180 min) resulted in enzyme instability and heightened juice viscosity, potentially obstructing the filtration process and diminishing clarity. This conclusion aligns with other research indicating that effective enzymatic hydrolysis necessitates sufficient reaction time to enhance efficiency while preventing enzyme degradation or unfavorable viscosity alterations [3, 5].

Using the BBD, pectinase and cellulase concentrations were evaluated at 0.1%, 0.3%, and 0.5%; incubation temperatures at 40°C, 50°C, and 60°C; and incubation times at 60, 120, and 180 min. Response surface analysis and desirability function optimization identified the optimal extraction conditions as 0.39% pectinase, 0.46% cellulase, an incubation temperature of 49.8°C, and an incubation time of 129.6 min. Under these conditions, juice yield reached 72.9%, while SSR was 23.1%, demonstrating a substantial improvement in extraction efficiency. The optimized process was evaluated by second‐order polynomial regression models for juice yield (Equation 5) and SSR (Equation 6), demonstrating high prediction accuracy (*R* ^2^ = 0.980 and 0.979, respectively).

(5) Yjuice yield=71.41.0084.5251.1670.96.4623.4888.658.42.6253.051.475+x1+x2+x3+x4−x12−x22−x32−x42+x1x2−x1x3+x3x4.

(6) YSSR=22.530.4831.40.4330.5331.8120.9882.8382.8881.2250.575+x1+x2+x3+x4−x12−x22−x32−x42+x1x2−x1x3+x1x4.

These findings are consistent with recent research on the enzymatic hydrolysis of fruit juices, in which pectinase increased pectin breakdown, lowering viscosity and increasing juice extraction efficiency [4, 5]. Similar enzymatic mechanisms have been reported in sapodilla juice [5] and carrot pulp hydrolysis [3], where optimal enzyme treatments resulted in higher yields and shorter processing times. Furthermore, Laskar et al. [6] found that combining enzymatic hydrolysis with hot water and citric acid pretreatments considerably increased banana juice extraction efficiency. The findings show that the synergistic effects of pectinase and cellulase, together with the optimum temperature and incubation period, are critical for increasing juice yield and SSR while preserving enzyme activity. The similarity in the response surfaces of juice yield and SSR can be attributed to the shared enzymatic extraction process, which influences both parameters using the same independent variables, such as pectinase and cellulase concentrations, incubation temperature, and duration. Enzymatic hydrolysis decreases juice viscosity by breaking down pectin‐rich structures and cellulose, making it easier to release juice and soluble particles at the same time. The synergistic impact displays similar trends and interaction patterns in the response surfaces of juice yield and SSR, emphasizing the role of enzyme concentration and incubation conditions in improving extraction efficiency and product quality. The findings highlight the importance of enzymatic hydrolysis in fruit juice and syrup production, providing an effective approach to improve processing efficiency, enhance product quality, and promote sustainable food production.

### 3.2. Verification of Optimized Process Performance

To validate the optimization model, experiments were conducted under the predicted optimal conditions: 0.39% pectinase, 0.46% cellulase, 49.8°C incubation temperature, and 129.6 min incubation time. The experimental results showed 71.4*%* ± 1.5*%* juice yield and 22.3*%* ± 0.9*%* SSR, which were only 2.1% and 3.5% lower than the predicted values (72.9% and 23.1%, respectively). These deviations, falling within an acceptable 5% range, confirm the model′s robustness and reliability. This optimization approach considers the synergistic actions of pectinase and cellulase in breaking down banana cell wall components, facilitating juice release along with other soluble constituents such as proteins and polysaccharides [20]. In addition to yield and SSR, the banana juice obtained had a TSS value of 15.8 ± 0.2°Brix and a pH of 4.4 ± 0.1.

A comparative evaluation with key experimental runs was performed. These included Run 5, Run 16, Run 20, and Run 24, and the results further support the model′s validity. Runs 24, 16, and 20, all applying the same conditions (0.3% pectinase, 0.3% cellulase, 50°C, and 120 min), produced consistent juice yields (71.0%–72.0%) and SSR (22.3%–22.7%), demonstrating reproducibility. Run 5, with the highest enzyme dosages (0.5% pectinase and 0.5% cellulase), gave the highest SSR (23.4%) but a slightly lower juice yield (70.4%), suggesting diminishing returns at elevated enzyme levels. Although the enzyme concentrations used in Run 24 were lower than those of the RSM‐predicted optimum, the slight advantage in enzyme savings must be weighed against the benefits of a statistically validated condition. The RSM optimum condition not only yielded the highest theoretical response but also offered a robust operational zone that can tolerate minor fluctuations in enzyme activity and processing conditions. This stability offers not only statistical support but also practical flexibility for industrial‐scale applications, as it allows for minor operational deviations without compromising performance [20–22]. Even small improvements in juice yield or SSR, such as 1%–2%, can lead to meaningful gains in large‐scale operations, which supports the adoption of the model‐derived condition.

It should be noted, however, that the minor discrepancies between predicted and actual values may stem from biological variability in raw material composition, enzyme activity, or local thermal conditions during incubation, which are common challenges in enzymatic extraction processes involving heterogeneous matrices such as banana pulp [23, 24]. Within this plateau region, further increases in enzyme loading or incubation time result in marginal gains or reduced efficiency, as widely reported in enzyme‐assisted extraction systems [21, 25]. The close agreement between predicted and observed values confirms the model′s accuracy and supports its role as a robust tool for guiding process optimization in enzymatic juice extraction.

### 3.3. Evaporation Process Optimization for High‐Quality Banana Syrup

The physicochemical properties of banana syrup were analyzed under different evaporation conditions, considering vital criteria including the yield of the syrup, TSS (°Brix), clarity, color parameters, acidity, pH, and processing time. The investigation was conducted under varying evaporation temperatures (40°C, 50°C, and 60°C) and vacuum levels (0, 60, 80, and 100 kPa gauge) while maintaining a constant water removal of 60%. The experimental results can be seen in Table 3, incorporating Duncan′s multiple range test groupings and standard deviations. The table allows comparisons to be drawn for the different parameters when the temperature and vacuum settings are systematically varied.

**Table 3:** Physicochemical properties of banana syrup under different evaporation conditions.

**Evap. temp (°C)**	**Vac. (kPa)**	**TSS (°Brix)**	**Clarity (%)**	**L** ^∗^	**a** ^∗^	**b** ^∗^	**Viscosity (mPa·s)**	**Process time (min)**
40	0	67.2 ± 0.8^c^	0.83 ± 0.19^bc^	78.2 ± 0.9^e^	6.2 ± 0.3^d^	86.4 ± 1.2^d^	146.6 ± 9.1^a^	181.4 ± 9.6^h^
	60	69.5 ± 0.4^fg^	0.88 ± 0.15^def^	79.3 ± 0.4^gh^	5.5 ± 0.3^abc^	87.2 ± 0.5^ef^	147.6 ± 9.9^a^	145.5 ± 6.2^e^
	80	70.1 ± 0.3^gh^	0.92 ± 0.17^f^	79.9 ± 0.6^hi^	5.1 ± 0.5^ab^	87.9 ± 0.4^fg^	150.9 ± 1.5^ab^	132.3 ± 9.9^c^
	100	71.2 ± 0.3^i^	0.88 ± 0.07^ef^	80.5 ± 0.4^i^	4.8 ± 0.4^a^	88.3 ± 0.8^g^	168.1 ± 9.6^bc^	121.7 ± 7.9^a^

50	0	66.1 ± 0.4^b^	0.81 ± 0.18^ab^	76.5 ± 0.9^bc^	7.3 ± 0.5^e^	84.5 ± 0.5^bc^	166.6 ± 6.1^bc^	195.6 ± 6.0^i^
	60	68.8 ± 0.4^de^	0.87 ± 0.16^cde^	78.6 ± 0.4^ef^	6.0 ± 0.2^cd^	86.0 ± 0.5^d^	166.0 ± 9.8^bc^	155.7 ± 8.4^f^
	80	69.6 ± 0.3^fgh^	0.91 ± 0.08^ef^	79.2 ± 0.4^fg^	5.6 ± 0.4^bcd^	86.8 ± 1.1^e^	181.5 ± 9.9^cd^	143.3 ± 9.0^d^
	100	70.5 ± 0.5^hi^	0.86 ± 0.26^cde^	79.8 ± 0.5^hi^	5.2 ± 0.5^abc^	87.5 ± 0.5^ef^	194.8 ± 5.4^de^	125.5 ± 7.1^b^

60	0	64.3 ± 0.3^a^	0.78 ± 0.24^a^	72.4 ± 0.6^a^	9.2 ± 0.2^g^	81.3 ± 0.5^a^	187.5 ± 7.1^d^	221.3 ± 8.4^j^
	60	67.9 ± 0.4^cd^	0.84 ± 0.14^bcd^	75.8 ± 0.7^b^	7.8 ± 0.4^f^	83.9 ± 0.5^b^	184.7 ± 6.3^cd^	182.3 ± 9.0^h^
	80	68.5 ± 0.6^de^	0.88 ± 0.11^def^	76.5 ± 0.4^c^	7.3 ± 0.4^e^	84.7 ± 0.4^c^	210.7 ± 7.4^ef^	164.6 ± 9.8^g^
	100	69.4 ± 0.3^ef^	0.90 ± 0.08^ef^	77.3 ± 0.9^d^	6.9 ± 0.5^e^	85.2 ± 0.5^d^	224.7 ± 5.7^f^	145.7 ± 7.5^e^

*Note:* Superscript letters for values in the same column are indicative of a lack of mutually significant difference (*p* ≥ 0.05).

#### 3.3.1. Effect of Evaporation Conditions on TSS

TSS (°Brix) rose with vacuum levels, with the greatest value (71.2 ± 0.3°Brix) achieved for 40°C and 100‐kPa gauge, while the minimum (64.3 ± 0.3°Brix) was achieved with no vacuum at 60°C. It can thus be stated that the preservation of soluble components can more readily be achieved via vacuum‐assisted evaporation. This approach ensures that sugar degradation to excess does not occur, and the final syrup will be more highly concentrated [9, 10]. In contrast, higher temperatures under atmospheric conditions (no vacuum) resulted in greater losses of volatile compounds and increased thermal degradation of sugars, leading to lower TSS values. Vacuum‐assisted evaporation at 80–100 kPa gauge minimized sugar degradation and enhanced the retention of soluble solids, resulting in a more concentrated syrup with improved quality [26, 27]. Data analysis conducted through the application of Duncan′s test (*p* < 0.05) confirmed that the highest TSS values in vacuum conditions (80–100 kPa gauge) were significantly different from those under lower vacuum or atmospheric conditions. This highlights the benefits of vacuum‐assisted evaporation in preserving soluble components while preventing excessive sugar degradation.

#### 3.3.2. Effect of Evaporation Conditions on Clarity

Clarity is vital in determining syrup quality. This parameter can be measured in the form of percentage transmittance at 670 nm, and the resulting value can indicate the extent of turbidity as well as the suspended solids content of the syrup. The findings demonstrated that evaporation temperature and vacuum levels have a significant influence upon clarity. The highest clarity (92.1*%* ± 0.7*%*) was recorded at 40°C with 80 kPa gauge, while the lowest (81.3*%* ± 1.8*%*) was observed at 60°C without vacuum (0 kPa gauge). High temperatures and nonvacuum conditions likely caused increased degradation of polysaccharides and protein denaturation, leading to greater turbidity [26]. Conversely, vacuum‐assisted evaporation at 80–100 kPa gauge resulted in significantly higher clarity, likely due to gentler processing conditions that reduced thermal degradation and the precipitation of soluble solids [27]. Duncan′s test confirmed that clarity at 40°C with 80–100 kPa gauge was significantly higher than at 50°C–60°C without vacuum, reinforcing the impact of mild processing on maintaining transparency.

#### 3.3.3. Effect of Evaporation Conditions on Color

The various different treatments resulting in significantly varying color outcomes, with the values for *L* ^*^ (lightness) maximized for 40°C and 100 kPa gauge (80.5 ± 0.4) and minimized for 60°C in the absence of vacuum (77.7 ± 2.4). Since *L* ^*^ declines with rising temperature and no vacuum, it can be inferred that intensified Maillard reactions take place along with caramelization, resulting in the production of a much darker syrup [28]. In conducting Duncan′s test, high‐vacuum conditions (80–100 kPa gauge) at temperatures of 40°C–50°C can be grouped together, with the outcomes suggesting superior color retention in contrast to the results at high temperatures with no vacuum [27]. The values for redness (*a* ^∗^) also rose significantly with no vacuum, with the maximum reached at 9.2 ± 0.2 at 60°C with no vacuum. This result is in accordance with the anticipated Maillard browning and sugar degradation which occurs at high temperatures, producing a darker color which is considered unappealing [26].

#### 3.3.4. Effect of Evaporation Conditions on Viscosity

The viscosity of banana syrup was also influenced by evaporation conditions. At 40°C with 100 kPa gauge, the syrup exhibited moderate viscosity (168.1 ± 9.6 mPa·s), which allowed efficient mass transfer and evaporation. However, at 50°C and 100 kPa gauge, viscosity increased significantly (194.8 ± 5.4 mPa·s) due to intensified sugar concentration, potentially reducing the efficiency of heat and mass transfer [29]. The highest viscosity (224.7 ± 5.7 mPa·s) was observed at 60°C with 100 kPa gauge, which likely contributed to slower evaporation rates despite the increased temperature. These results are in concurrence with those of earlier works and suggest that a rise in the solute concentration during the course of the evaporation process serves to increase viscosity, in turn causing lower heat transfer efficiency and reducing the rate of mass transfer [30]. Moderate vacuum levels (80–100 kPa gauge) and lower temperatures (40°C–50°C) strike a balance between maintaining a manageable viscosity and maximizing evaporation efficiency [31].

#### 3.3.5. Effect of Evaporation Conditions on Processing Time

Processing time was strongly influenced by vacuum level and temperature. The shortest processing time (121.7 ± 7.9 min) was recorded at 40°C with 100 kPa gauge, whereas the longest time (221.3 ± 8.4 min) was observed at 60°C without a vacuum. The faster evaporation rate at 40°C with 100 kPa gauge may be attributed to an optimal balance between vacuum pressure and heat transfer efficiency. In line with Dak et al. [32], at higher temperatures, such as 50°C and 60°C with 100 kPa gauge, heat‐induced viscosity changes and increased thermal degradation may have contributed to slower evaporation rates due to reduced mass transfer efficiency. These findings concur with earlier studies, in which it was found that a greater solute concentration leads to increased viscosity, thus reducing evaporation rates due to hindered thermal conductivity and limited mass transport [33].

### 3.4. Optimization of Vacuum Evaporation for Banana Syrup Production

The vacuum‐assisted evaporation process was optimized to preserve the physicochemical properties of banana syrup while enhancing concentration efficiency. Among the tested conditions, vacuum evaporation at 40°C under 100 kPa vacuum pressure was found to be the most effective, 71.2 ± 0.3°Brix TSS, and superior clarity (0.92*%* ± 0.07*%*). Compared to conventional atmospheric evaporation, vacuum processing significantly reduced enzymatic browning and caramelization, leading to better color retention and reduced viscosity. The reduction in browning can be attributed to lower oxygen availability and minimal heat exposure under vacuum conditions, preserving natural pigments and bioactive compounds [9, 10]. Similar observations were reported in studies on pomegranate juice concentration, where rotary vacuum evaporation minimized color degradation and maintained sensory quality better than traditional heating [10]. Additionally, vacuum microwave evaporation of apple juice resulted in superior retention of phenolic compounds, indicating that vacuum‐assisted processing effectively reduces heat‐induced degradation [9]. The viscosity of banana syrup increased with prolonged evaporation, particularly at higher temperatures (50°C–60°C), due to the concentration of soluble solids and sugar components. However, at 40°C and 100 kPa vacuum, viscosity remained within an acceptable range, facilitating efficient mass transfer without excessive thickening. In order to achieve ideal syrup flow characteristics, it is necessary to achieve the correct balance among the processing factors of time, temperature, and vacuum pressure. This is important because high viscosity is undesirable since there is a tendency for evaporation efficiency to decrease [29, 30].

Further optimization identified that vacuum‐assisted evaporation at moderate vacuum levels (80–100 kPa gauge) combined with lower temperatures (40°C–50°C) provided the best balance between process efficiency and product quality. These conditions maximized syrup yield while maintaining desirable physicochemical properties. Among the tested conditions, evaporation at 40°C with 100 kPa gauge yielded the best results, retaining the highest TSS (71.2 ± 0.3°Brix) and maintaining excellent clarity (0.92*%* ± 0.07*%*). Furthermore, the syrup viscosity remained at a manageable level (168.1 ± 9.6 mPa·s), allowing for efficient processing. Furthermore, there was a significant decline in processing time to just 121.7 ± 7.9 min, from which it can be inferred that vacuum‐assisted evaporation is effective in boosting evaporation rates while mitigating the problem of thermal degradation. However, when evaporation is performed at greater temperatures, such as 60°C, with no vacuum, the quality becomes significantly lower; this was observed in syrup that underwent excessive browning and greater viscosity, while the processing time was significantly longer, at 221.3 ± 8.4 min. Processing without a vacuum was also shown to reduce clarity and increase thermal degradation, having an adverse influence upon the quality of the final product. Accordingly, it can be confirmed that vacuum‐assisted evaporation is vital in enhancing both the quality and efficiency of banana syrup production. The approach leads to a shorter processing time while ensuring that clarity, viscosity, and color are maintained at desirable levels, ensuring a sustainable and efficient process. Moderate vacuum levels (80–100 kPa gauge) and relatively low temperatures (40°C–50°C) allow syrup production to be carried out effectively to deliver a product of acceptable quality.

## 4. Conclusion

This study successfully optimized the enzymatic extraction and vacuum evaporation process for banana syrup production, improving both efficiency and product quality. The optimized enzymatic conditions (0.39% pectinase, 0.46% cellulase, 49.8°C incubation temperature, and 129.6 min) yielded 71.4*%* ± 1.5*%* juice and 22.3*%* ± 0.9*%* SSR, with minimal deviation from the predicted values (2.1% and 3.5% error, respectively). Vacuum‐assisted evaporation at 40°C under 100 kPa was identified as the optimal condition, preserving color, clarity, and viscosity while minimizing processing time.

## Ethics Statement

Ethical approval was not required for this study.

## Conflicts of Interest

The authors declare no conflicts of interest.

## Author Contributions

Narathip Sujinda: conceptualization (equal), data curation, formal analysis, investigation, methodology, validation, visualization, writing—original draft, and writing—review and editing. Natthiya Chaichana: conceptualization (equal), data curation, funding acquisition, project administration, and supervision. Thanapon Saengsuwan: conceptualization (equal), data curation, funding acquisition, resources, and supervision. Jaturapatr Varith: resources.

## Funding

No funding was received for this manuscript.

## Data Availability

The data that support the findings of this study are available from the corresponding author upon reasonable request.
